# *AaERF1* Positively Regulates the Resistance to *Botrytis cinerea* in *Artemisia annua*

**DOI:** 10.1371/journal.pone.0057657

**Published:** 2013-02-28

**Authors:** Xu Lu, Weimin Jiang, Ling Zhang, Fei Zhang, Fangyuan Zhang, Qian Shen, Guofeng Wang, Kexuan Tang

**Affiliations:** Plant Biotechnology Research Center, Fudan-SJTU-Nottingham Plant Biotechnology R&D Center, School of Agriculture and Biology, Shanghai Jiao Tong University, Shanghai, People’s Republic of China; University of the West of England, United Kingdom

## Abstract

Plants are sessile organisms, and they can not move away under abiotic or biotic stresses. Thus plants have evolved a set of genes that response to adverse environment to modulate gene expression. In this study, we characterized and functionally studied an ERF transcription factor from *Artemisia annua*, *AaERF1*, which plays an important role in biotic stress responses. The *AaERF1* promoter had been cloned and GUS staining results of *AaERF1* promoter-GUS transgenic *A. annua* showed that *AaERF1* is expressed ubiquitiously in all organs. Several putative *cis*-acting elements such as W-box, TGA-box and Py-rich element, which are involved in defense responsiveness, are present in the promoter. The expression of *AaERF1* can be induced vigorously by methyl jasmonate as well as by ethephon and wounding, implying that *AaERF1* may activate some of the defense genes via the jasmonic acid and ethylene signaling pathways of *A. annua*. The results of electrophoretic mobility shift assay (EMSA) and yeast one-hybrid experiments showed that *AaERF1* was able to bind to the GCC box *cis*-acting element *in vitro* and in yeast. Ectopic expression of *AaERF1* could enhance the expression levels of the defense marker genes *PLANT DEFENSIN1.2* (*PDF1.2*) and *BASIC CHITINASE* (*ChiB*), and increase the resistance to *Botrytis cinerea* in the 35S::*AaERF1* transgenic Arabidopsis. The down-regulated expression level of *AaERF1* evidently reduced the resistance to *B. cinerea* in *A. annua*. The overall results showed that *AaERF1* positively regulated the resistance to *B. cinerea* in *A. annua*.

## Introduction

The necrotrophic fungus *Botrytis cinerea* causes significant economic losses throughout the world as a destructive pathogen of a broad spectrum of plant species [1]. Plants are sessile, thus they have evolved some gene families to cope with pathogen attack through complex adaptive responses. The AP2/ERF transcription factors are one of the most important families that are involved in plant response to biotic and abiotic stresses as well as in the development of various plant species [2]. The AP2/ERF transcription factors, which have the conserved AP2/ERF transcription factors binding domains of 57–66 amino acids, constitute a large multigene family divided into five subfamilies named AP2, CBF/DREB, ERF, RAV and the fifth (comprising members not assigned to other four groups) [3], [4]. The AP2 subfamily proteins contain two repeated AP2/ERF domains, while the RAV family proteins contain a B3 domain and a single AP2/ERF domain. In contrast to the AP2 and RAV subfamily members, the CBF/DREB and ERF subfamily proteins contain single AP2/ERF domain [5]. The genes in the CBF/DREB subfamily play a crucial role in the resistance of plants to abiotic stresses by recognizing the dehydration responsive or cold-repeat element (DRE/CRT) with a core motif of A/GCCGAC [6]. The ERF subfamily is often involved in the response to plant stresses like pathogenesis by modulating the expression of their target genes via binding to the *cis*-acting element AGCCGCC, known as the GCC box in their promoters [5].

Jasmonic acid (JA) and ethylene (ET) are two important hormones that act synergistically during plant resistance to necrotrophic pathogens such as *B. cinerea* by activating some ERF genes, which are responsive to both JA and ET treatment. For example, in Arabidopsis, two ERF genes, *ERF1* and *ORA59*, are induced by JA and ET [7], [8]. Overexpression of either *ERF1* or *ORA59* resulted in constitutive expression of the defense marker genes, *PLANT DEFENSIN1.2* (*PDF1.2*) and *BASIC CHITINASE* (*B-CHI*), thus enhancing the resistance to *B. cinerea* [7], [8]. A recent study also showed that another two ERF genes, *ERF5* and *ERF6*, play redundant roles as positive regulators of JA/ET-mediated defense against *B. cinerea* in Arabidopsis [9]. Thus, at least four ERFs play key role in integrating the JA and ET signal in disease resistance. In previous studies, our group also showed that two ERFs from *Gossypium barbadense*, showed positive effects on disease resistance [10], [11]. ERF genes from other species, such as tomato (*TSRF1* and *Pti4*), soybean (*GmERF3*) and *Bupleurum kaoi* (*BkERF1*, *BkERF2.1* and *BkERF2.2*) also showed positive effects on disease resistance, suggesting the ERF genes have a conserved role in diverse species to counteract with plant pathogens [5], [12]–[16].

*Artemisia annua* L. is an important medicinal plant that produces artemisinin, which is widely used in malaria treatment. A recent study has shown that JA can increase artemisinin production by inducing two ERF genes in *A. annua*. AaERF1 and AaERF2, both of which directly bind to the CRTDREHVCBF2 (CBF2) and RAV1AAT (RAA) motifs present in both *ADS* and *CYP71AV1* promoters to activate those key enzymes in artemisinin biosynthesis pathway [17]. Our previous study showed that wounding stress also significantly elevated the artemisinin content by increasing *ADS* and *CYP71AV1* expression levels [18]. Compared to the great effort in artemisinin biosynthesis pathway, little is known about the disease resistance in *A. annua*. Since ERFs are key regulators that integrate JA and ET signals in disease resistance, it is attempted to establish whether *AaERF1* has a role in disease resistance. Thus, our research focused on the function of *AaERF1* in plant antifungal field and illustrated that *AaERF1*, which could bind to GCC box in *in vitro* and in yeast, positively regulated the resistance to *B. cinerea* in *A. annua*.

## Results

### AaERF1 is Ubiquitously Expressed in *A. annua*

**Figure 1:**
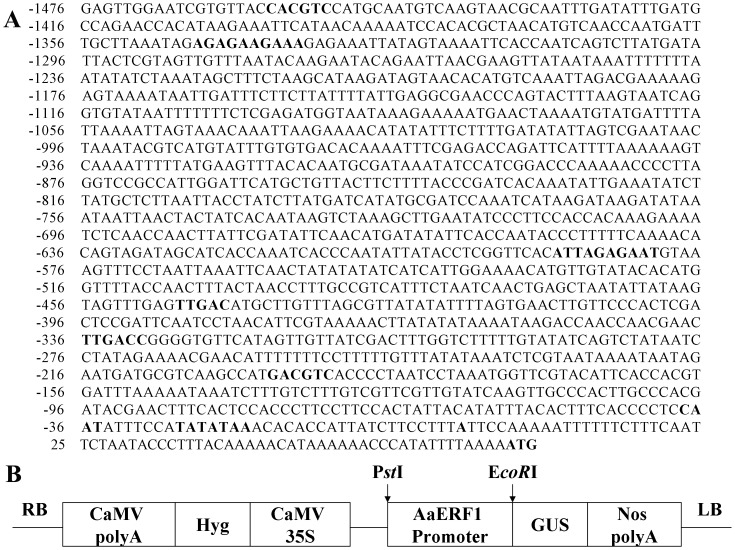
Sequence of *AaERF1* promoter region and construction of *AaERF1* promoter-GUS vector. (A) The sequence of *AaERF1* promoter region. The transcription initiation site and the translation start site are in bold, underlined letters. Numbers indicate the position relative to the transcription start site. Putative *cis*-acting regulatory elements involved in defense responsiveness in *AaERF1* promoter are in bold. (B) Construction of *AaERF1* promoter-GUS vector. *Pst*I and *Eco*RI are the enzymes used in the construction.

**Figure 2:**
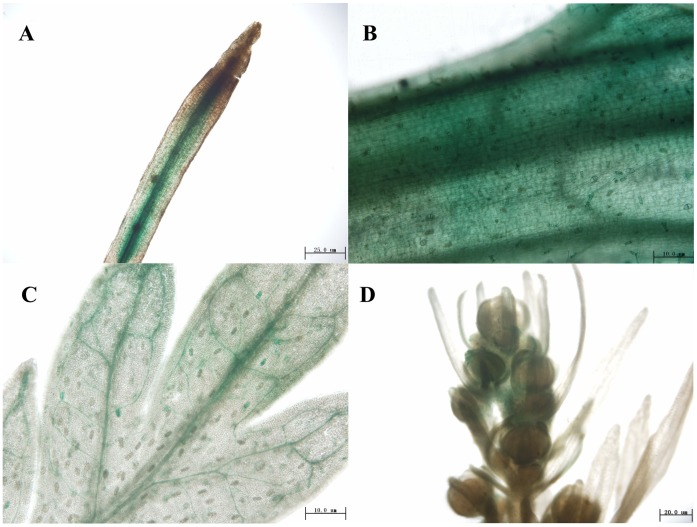
Localization of *AaERF1* expression using GUS staining of promoter:GUS transgenic plants. GUS activity is revealed by histochemical staining. (A) Root. (B) Stem. (C) Leaf. (D) Flower buds.

The promoter sequence of *AaERF1*(JQ513909)was cloned by genomic walking (Figure 1A). To observe the expression pattern of *AaERF1* in details, the *AaERF1* promoter was subcloned to the pCAMBIA1391Z vector (Figure 1B) and then *AaERF1* promoter-GUS transgenic *A. annua* plants were generated. Six lines of the transgenic *A. annua* plants expressing the GUS and three lines for the wild-type background were prepared. All the lines showed similar fusion protein expression. GUS activity was detected in all tissues examined, including roots, stems, leaves and flowers (Figure 2A, 2B, 2C and 2D). In 1-month-old plants, GUS activity was high in root tips, stems and leaves (Figure 2A, 2B and 2C). During the flowering period, GUS activity was also detected in flower buds. So, *AaERF1* is ubiquitously expressed in *A. annua*. From Figure 2B and 2C, GUS expression was also detected in the glandular trichomes and T-shaped trichomes. No signals were observed in the negative control plants transformed with pCAMBIA1391 empty vector (Figure S1).

### Prediction of *cis*-acting Elements of Promoter Region of *AaERF1*

Putative *cis*-acting elements of the promoter were predicted using the PLANTCARE software (http://bioinformatics.psb.ugent.be/webtools/plantcare/html/) (Figure 1A; Table 1). A putative TATA box sequence was found at -27 bp, and the putative CAAT box sequence was located at -38 bp. The 5′-UTR pyrimidine-rich stretch site is a *cis*-acting element conferring high transcription levels. Such an element was found at position -1345 to -1336 as shown in Figure 1A. A TC-rich repeat, which is involved in defense and stress response, was localized to position -590 to -581. A TGA-box element (TGACGTCA), which is involved in plant defense responsiveness, was found at position -209 to -201. A G/C-box element (CACGTC), which is involved in light-induction or hormone control, was found at position -1458 to -1453. The W box is a fungal elicitor responsive element, which was present at positions -547 to -542 bp and -336 to -332 bp in *AaERF1* promoter. A search for the regulatory elements in *AaERF1* promoter also carried EIRE box. The above *cis*-acting elements are summarized in Table 1. Nearly all these *cis*-acting elements are related to defense responsiveness. Consequently, *AaERF1* may be a defense responsiveness transcription factor in *A. annua*.

**Table 1:** Putative *cis*-acting regulatory elements involved in defense responsiveness in *AaERF1* promoter.

*Cis*-elements	Motif and position	Putative function
5-UTR pyrimidine-rich stretch consensus:TTTCTTCTCT	−1345 AGAGAAGAAA -1336	*cis*-acting element conferring high transcription
EIRE-box: TTGACC	−336 TTGACC -331	elicitor responsive element
W-box consensus: TTGAC	−547 TTGAC -542; -336 TTGAC -332	fungal elicitor responsive element
TGA-box: TGACGTCA	−209 TGACGTCA -201	*cis*-acting element conferring plant defense responsiveness
G/C-box consensus: CACGTC	−1458 CACGTC -1453	*cis*-acting element involved in light-induction or hormone control
TC-rich repeats: ATTTTCTTCA	−590 ATTAGAGAAT -581	*cis*-acting element involved in defense and stress responsiveness

### Expression Profiling Analysis of *AaERF1* after Hormone and Stress Treatments

**Figure 3:**
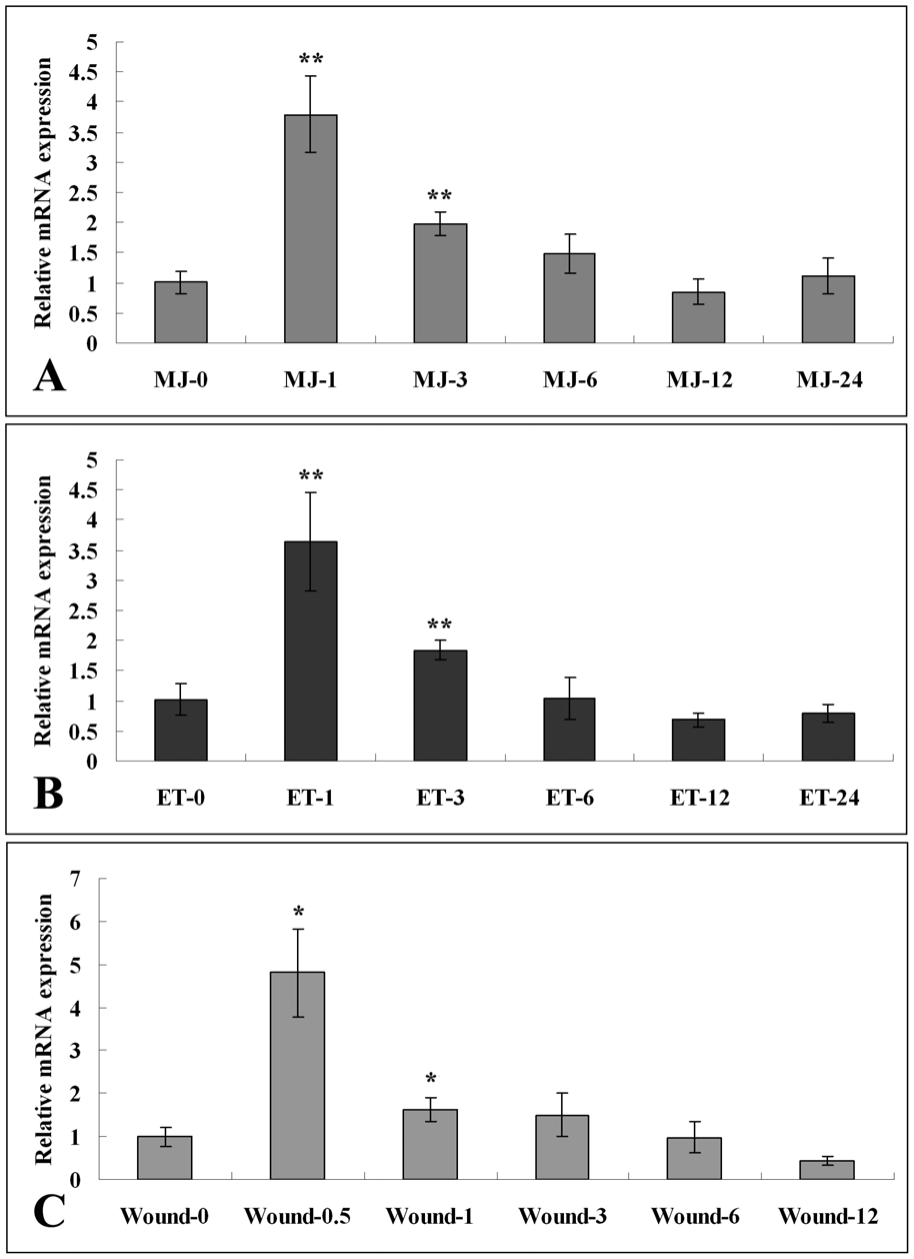
Expression patterns of *AaERF1* in response to hormone and stress treatments by RT-Q-PCR. A. Relative expression levels of *AaERF1* after MeJA (100 µM). B. Relative expression levels of *AaERF1* after ethephon (500 µM). C. Relative expression levels of *AaERF1* after wound treatment. Total RNA was isolated respectively from *A. annua* leaves under different treatments for different periods of time (0 h, 0.5 h, 1 h, 3 h, 6 h, 12 h and 24 h) followed by RT-Q-PCR analysis with the gene-specific primers AaERF1-RT-F and AaERF1*-*RT-R. Values indicate the mean fold relative to sample 0 h. *Actin* is used as a control for normalization. Data are averages ± SE from three independent experiments.

In this study, RT-Q-PCR analysis was used to obtain the expression pattern of *AaERF1* after hormone and stress treatments including MeJA (100 µM), ethephon (500 µM) and wound treatments. The transcript level of *AaERF1* was increased rapidly and peaked within 1 h after MeJA treatment, followed by a gradually decline (Figure 3A). The treatment with ethephon shows a similar expression pattern with the treatment of MeJA (Figure 3B). The transcript level of *AaERF1* was also sensitive to stress treatments. Wounding could induce a significant accumulation of *AaERF1* transcript in a short time period (0.5 h). Then the transcript level was quickly decreased (Figure 3C). The statistics analysis showed that the observed differences were statistically significant.

### Comparative and Bioinformatic Analyses of *AaERF1*

The results of the BLAST-Protein (BLASTP) online (http://www.ncbi.nlm.gov/blast) showed that the AaERF1 protein had a highly conserved AP2 domain with other ERF proteins, including Arabidopsis AtERF1, AtERF2, ORCA3, LeERF1, NtERF1, TaERF3 and ORA59 (Figure S2A). This domain is divided into two conserved segments of YRG and RAYG, in which a β-sheet and α-helix are predicted (β-α motif; see Figure S2A). A phylogenetic tree of ERF proteins was drawn using the CLUSTAL X program. The phylogenetic tree demonstrated that ERF proteins originated from a common ancestor and diverged into several groups (Figure S2B). According to the phylogenetic tree, the protein of AaERF1 had close evolutionary relationships to AtERF2, LeERF1, NtERF1 and TaERF3 which showed that they might share similar functions in disease resistance (Figure S2B).

### AaERF1 Protein Interacts with the GCC Box *in vitro*

**Figure 4:**
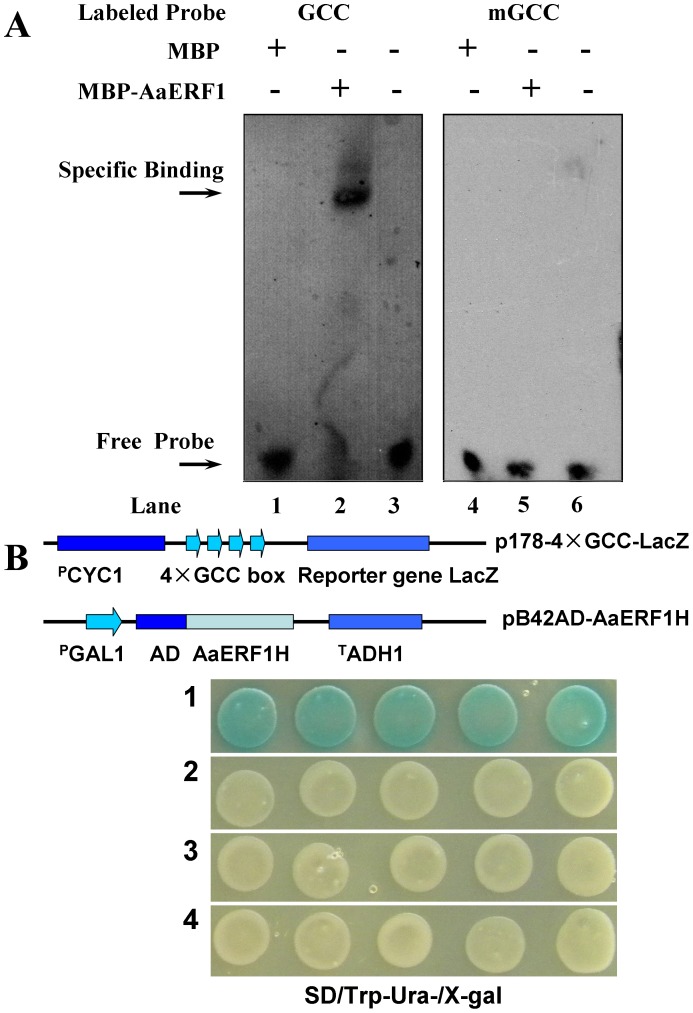
The DNA binding ability of AaERF1 via GCC box. A. Electrophoretic mobility shift assays on DNA binding of AaERF1. Lane 1: negative controls contain MBP plus labelled GCC probe; lane 2: the MBP–AaERF1 protein plus labelled GCC probe; lane 3: only labelled GCC probe; lane 4: negative controls contain MBP plus labelled mutated GCC probe; lane 5: MBP–AaERF1 plus labelled mutated GCC probe; lane 6: only labelled mutated GCC probe. The protein–GCC probe complex and free probes are indicated respectively by two arrows. B. GCC box binding analysis of AaERF1 using the yeast one-hybrid system. Sketch maps show the construction of vectors used in this experiment. Photographs show the growth behavior of transformants on SD/Trp−Ura−/X-gal medium. Sector 1: p178-4×GCC-LacZ+pB42AD-AaERF1; sector 2: p178+ pB42AD-AaERF1; sector 3: p178-4×GCC-LacZ+pB42AD; sector 4: p178+ pB42AD.

Since the AP2 domain of *AaERF1* contained the key amino acids to bind the GCC box, the recombinant MBP-AaERF1 protein was constructed and overexpressed in *E. coli* BL21, purified, and used to examine the DNA binding ability *in vitro*. The purified MBP-AaERF1 protein was mixed, respectively, with the labeled wild-type GCC probe or a mutated GCC probe in the binding reaction. The results of EMSA showed that the gel mobility shift was specific to the MBP-AaERF1 protein with the labeled GCC probe (lane 2 in Figure 4A). As expected, there were no shifted bands in the combination of MBP-AaERF1 plus the mutated GCC (mGCC) probe (lane 5 in Figure 4A) and in the negative controls, including MBP with the labeled GCC probe (lane 1) or mGCC probe (lane 4), and only the labeled GCC probe (lane 3) or mGCC probe (lane 6) (Figure 4A). The results demonstrated that AaERF1 was able to bind to the GCC box *cis*-acting element, but not to the mutated GCC box *in vitro*.

### AaERF1 can Bind to the GCC Box in Yeast

The yeast one-hybrid system is a stable system to study the DNA binding ability of transcription factors [19]. The results of yeast one-hybrid and β-galactosidase activity assays indicated that only the hybrid cells containing the combination of pB42AD::AaERF1 and p178-4×GCC-LacZ showed β-galactosidase activity compared with other combinations, including pB42AD with p178-LacZ, pB42AD::AaERF1 with p178-4×GCC-LacZ, pB42AD::AaERF1 with p178-LacZ, and pB42AD with p178-4×GCC-LacZ. The results demonstrated that AaERF1 could bind to the GCC box *cis*-acting element in yeast cells (Figure 4B).

### *AaERF1*-overexpression in Arabidopsis Causes the Increase of Disease Resistance to *B. cinerea*

**Figure 5:**
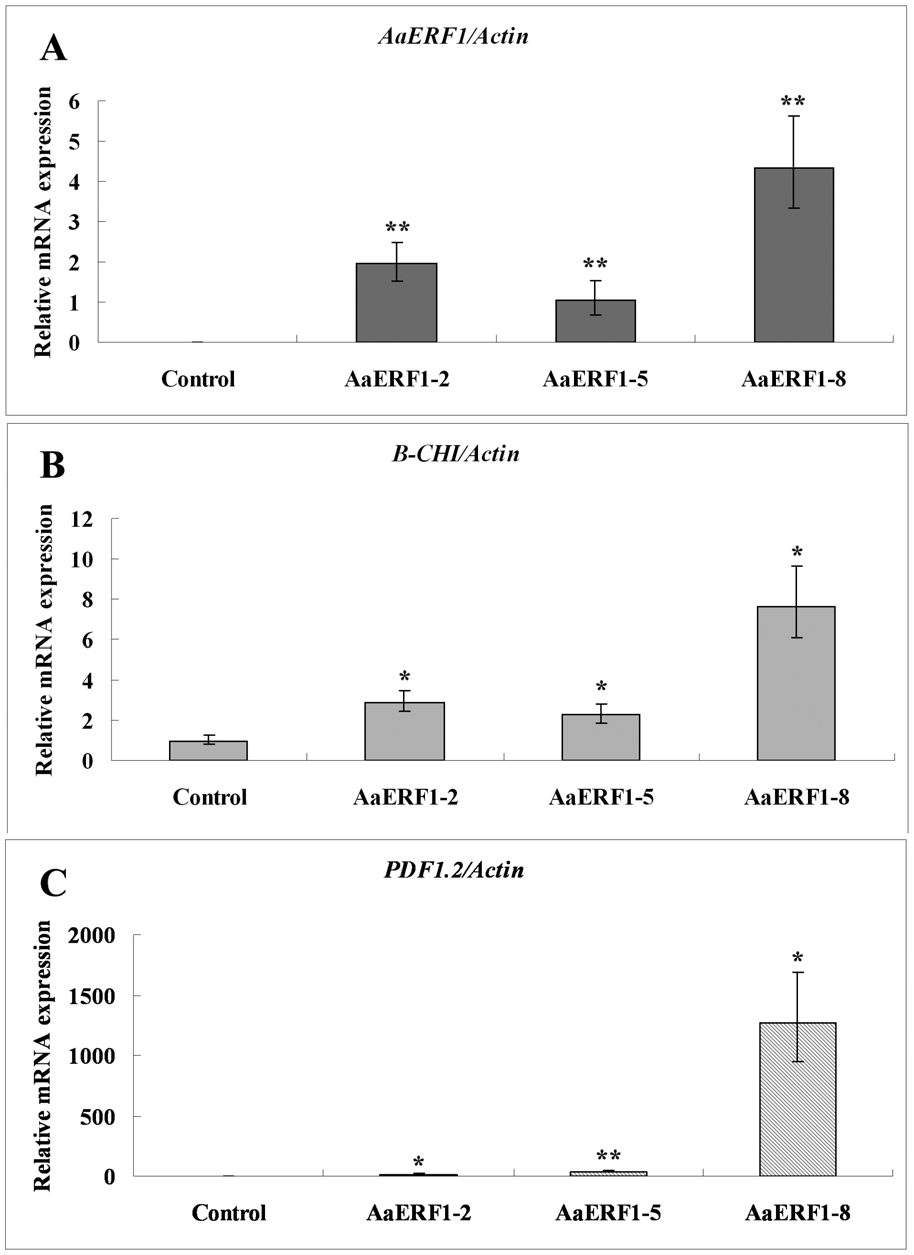
The expression levels of *AaERF1, Chi-B* and *PDF1.2* in 35S::*AaERF1* transgenic Arabidopsis analyzed by RT-Q-PCR. Vertical bars represent standard deviation. A. The expression of *AaERF1* in the control and transgenic Arabidopsis plants. Values indicate the mean fold relative to sample the AaERF1-5 transgenic plants. B. The expression of *Chi-B* in the control and transgenic Arabidopsis plants. Values indicate the mean fold relative to sample the pCAMBIA2300+ empty vector transgenic plants C. The expression of *PDF1.2* in the control and transgenic Arabidopsis plants. Values indicate the mean fold relative to sample the pCAMBIA2300+ empty vector transgenic plants. *Actin* is used as a control for normalization. Data are averages ± SE from three independent experiments.

The transgenic Arabidopsis plants were first confirmed by kanamycin-resistant screening and genomic DNA-based PCR, and then three transgenic lines were chosen for further analysis. The control experiment involving the transfer of empty plasmid p2300+ to Arabidopsis was also conducted. The results showed that the transcript levels of *AaERF1* had a significant increase in *AaERF1*-overexpression lines (Figure 5A). Correspondingly, *Chi-B* was shown to be elevated between 2.3- and 7.7-fold in *AaERF1*-overexpression lines (Figure 5B). The transcript levels of *PDF1.2* were elevated between 15- and 1269-fold than that of the control (Figure 5C). The statistics analysis showed that the observed differences were statistically significant.

**Figure 6:**
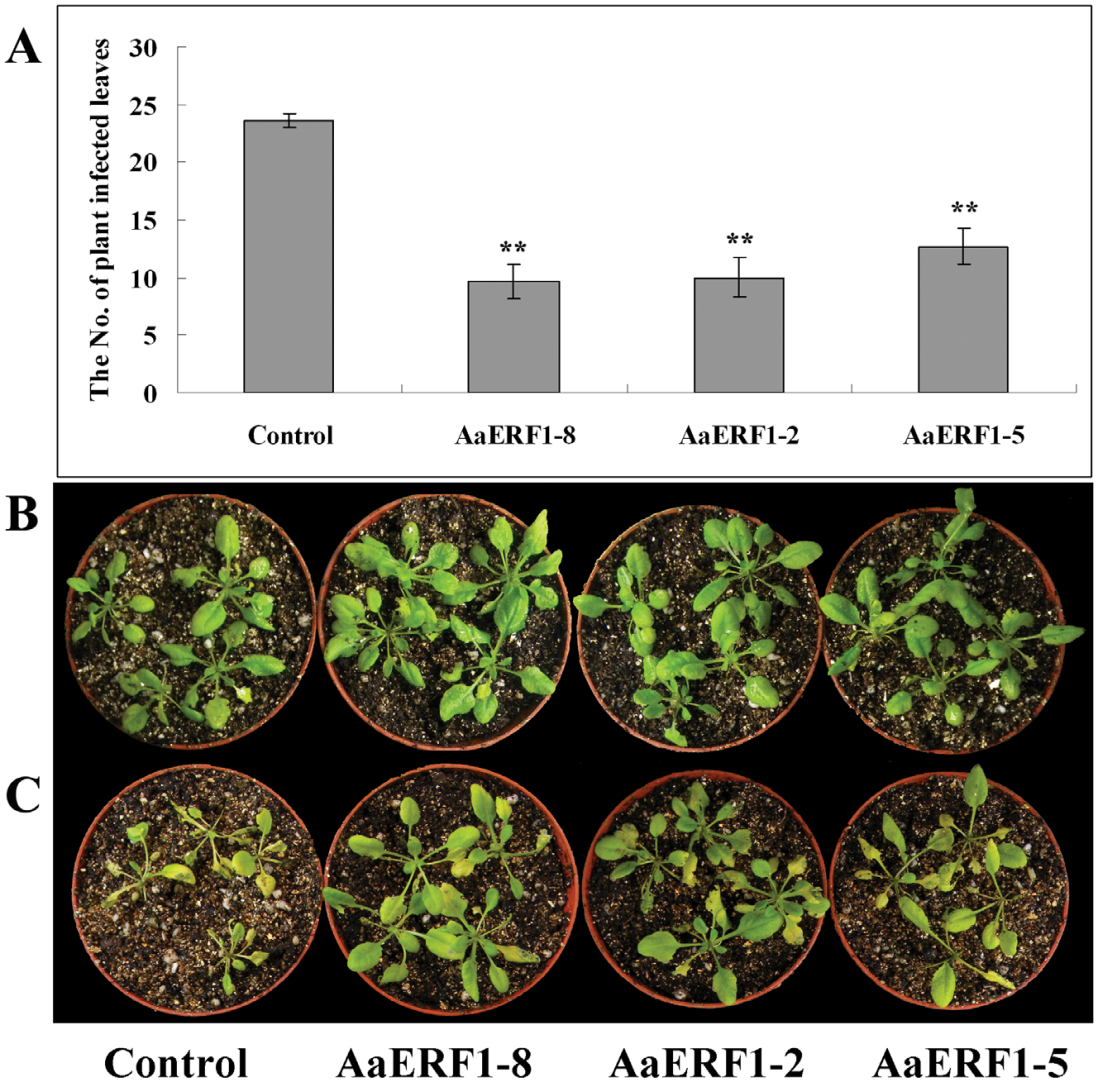
The 35S: *AaERF1* lines show increased disease resistance. A. The numbers of control and the three independent 35S: *AaERF1* transgenic Arabidopsis lines showing disease symptoms 4 d after inoculation with *Botrytis cinerea*. Average data with standard errors from three biological replicates are shown. B. The control and 35S: *AaERF1* lines, without inoculation with *Botrytis cinerea*. C. The control and 35S: *AaERF1*, 4 d after inoculation with *Botrytis cinerea*, with 35S: *AaERF1* plants showing reduced disease symptoms (see “Materials and Methods” for description).

The *AaERF1*-overexpression lines were observed following inoculation with *B. cinerea*. For each of the *AaERF1*-overexpression lines, we observed a significant reduction in the development of disease symptoms in independent inoculation experiments. Four days following inoculation with *B. cinerea*, 79% of the control plants showed symptoms of infection, whereas only between 32% and 42% of the leaves from *AaERF1*-overexpression lines were symptomatic (Figure 6A, 6C). The statistics analysis showed that the observed differences were statistically significant. The control plants turned dry and died, while most of the *AaERF1*-overexpression plants were growing well (Figure 6B, 6C). The results showed that the overexpression of *AaERF1* could increase the disease resistance to *B. cinerea* in Arabidopsis.

### Down-regulated Expression Level of *AaERF1* in *A. annua* Causes the Reduction of Disease Resistance to *B. cinerea*

**Figure 7:**
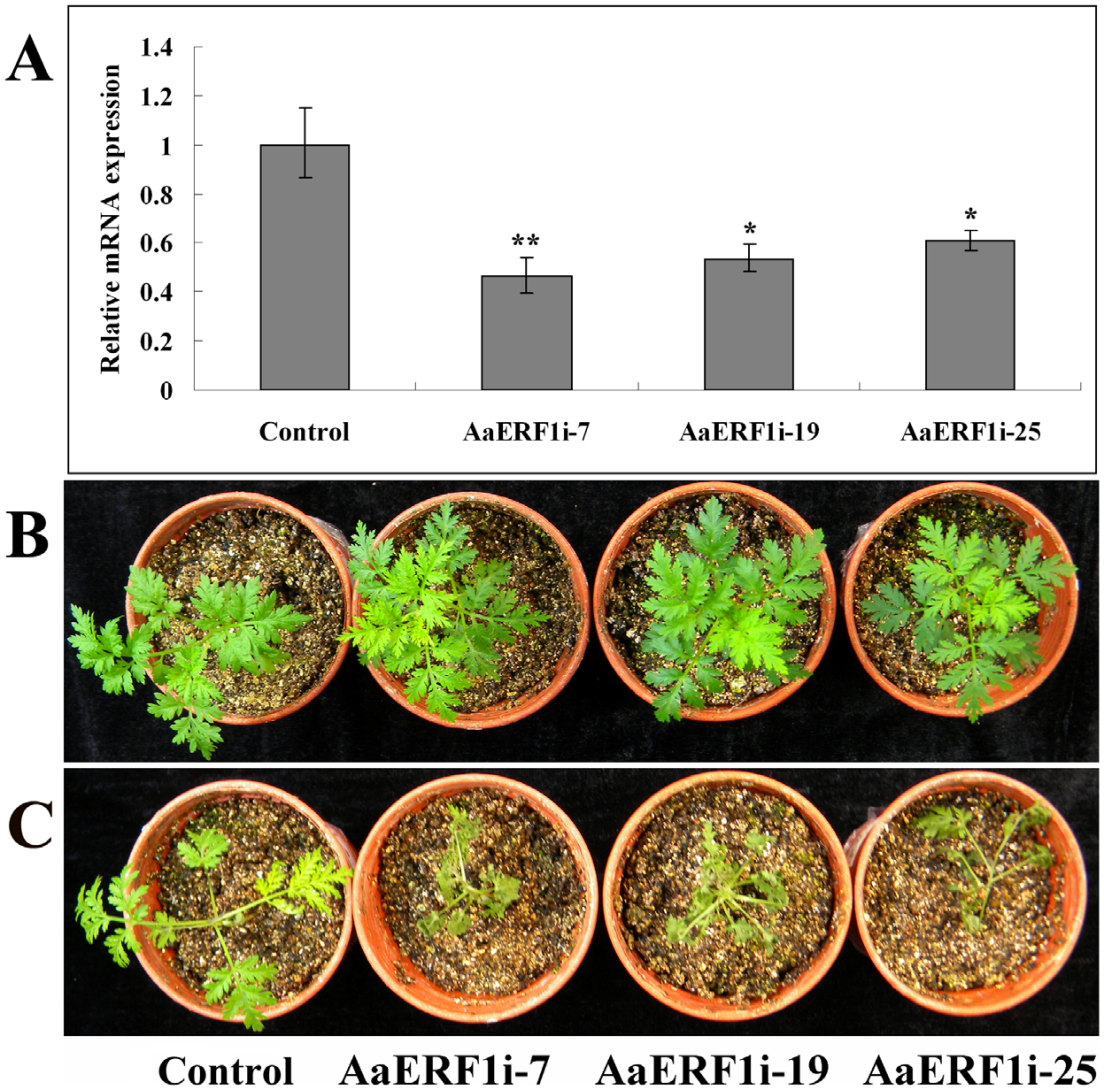
The RNAi lines of *AaERF1* show decreased disease resistance. A. The expression of *AaERF1* in the empty vector and *AaERF1i* transgenic *A. annua* plants. Error bars are SE (n = 3). B. The empty vector and *AaERF1i* lines, without inoculation with *Botrytis cinerea*. C. The empty vector and *AaERF1i* lines, 6 d after inoculation with *Botrytis cinerea*, with *AaERF1i* lines showing increased disease symptoms. The experiment was performed three times with similar results.

Here, we constructed the RNAi vector of *AaERF1* and transformed it into *A. annua*. The control experiment involving the transfer of empty plasmid pCAMBIA2300+ to *A. annua* was also conducted. The transgenic plants were first confirmed by genomic DNA-based PCR using the 35S forward primer, *AaERF1* reverse primer and the reverse primer of kanamycin-resistant gene (Figure S3), and then three independent transgenic lines were chosen for further analysis. In the RNAi transgenic lines, the transcript levels of *AaERF1* were suppressed to 46–61% of the control level (Figure 7A). The statistics analysis showed that the observed differences were statistically significant.

The three independent *AaERF1*i lines were inoculated with *B. cinerea*. The results showed that each of the *AaERF1*i lines had a significant reduction in the disease symptoms in three independent inoculations. Six days following inoculation with *B. cinerea*, most of the leaves in *AaERF1*i lines were dry and dead, while most of the the control plants were growing well (Figure 7B). The results showed that *AaERF1* was a positive regulator to the disease resistance to *B. cinerea* in *A. annua*.

## Discussion

The putative *cis*-acting elements of *AaERF1* promoter were predicted as shown in Figure1A and summarized in Table 1. The W box (TTGAC) is the binding site for members of the WRKY family of transcription factors [20]. The importance of W boxes was illustrated by studies on Arabidopsis transcription during systemic-acquired resistance [21]. Previous reports indicated that the G-box–related hexamers(CACNTG,CACATG and (T/C)ACGTG)are the binding sites of MYC2 [22]–[24]. MYC2 is a negative regulator of the JA-responsive pathogen defense genes *PDF1.2* and *B-CHI* [25]. At -209bp of *AaERF1* promoter, there is a TGA motif, which is a perfect binding site for TGA transcription factors. TGA transcription factors are essential for the activation of JA and ET dependent defense mechanisms in Arabidopsis [26]. In addition, we identified the TC-rich repeats (ATTTTCTCCA) in the promoter of *AaERF1*, which was previously described in tobacco (*Nicotiana tabacum*) as *cis*-acting elements involved in defence and stress responsiveness [27]. All above elements are related to the disease resistance, implying that *AaERF1* may have a similar function.

The results of RT-Q-PCR showed that the transcript level of *AaERF1* was increased rapidly and peaked within 1 h after MeJA, ethephon and wound treatments, followed by a gradual decline. Jasmonates and ethylene are considered the major signal compounds for wound-induced gene expression in plants [28]. Methyl jasmonate was reported as a volatile compound emitted from the leaves of *A. tridentata* subspecies *tridentata* resulting in the induction of defense-related genes in nearby tomato plants [29]. So, the transcript of *AaERF1* can be induced vigorously by MeJA, ethephon or wound treatments, implying that *AaERF1* may play an important role in the JA and ET signaling pathways and have some function in disease resistance of *A. annua*.

The bioinformatic analysis showed that the AP2/ERF domain of AaERF1 contained a β-sheet and α-helix (β-α motif; see Figure S2A), all of which are important for DNA binding with the GCC Box [30], [31]. The phylogenetic tree analysis of AaERF1 showed that AaERF1 had close relationship with AtERF2 and TaERF3. *AtERF2* and *TaERF3* have been well characterized and their functions were mainly related to disease resistance, at least in part, via binding to the GCC box in the promoter region of downstream genes [19], [32]–[34]. So, all above analysis implied that the protein of AaERF1 has a function in disease resistance and may have the GCC Box binding ability.

From the results of EMSA and yeast one-hybrid experiment, we know that AaERF1 was able to bind to the GCC box *cis*-acting element *in vitro* and in yeast cells. The ERF subfamily of proteins recognizes the *cis*-acting element GCC box, which is mainly involved in the response to biotic stresses like pathogenesis [5]. Enhancement of disease resistance in plants has been achieved by overexpressing ERF proteins, such as Arabidopsis *AtERF1* [8], [35], *AtERF2* [31] and rice *OsBIERF3* [36]. So, we infer that the overexpression of *AaERF1* could enhance the disease resistance in plants.

*PDF1.2* and *Chi-B* in Arabidopsis were marker genes of the resistance to several fungi, including *B. cinerea* [35], [37]. The results of RT-Q-PCR showed that the transcripts of *AaERF1*, *Chi-B* and *PDF1.2* showed an obvious correlated increase in *AaERF1*-overexpression lines, which were similar with the overexpression of *ORA59* in Arabadopsis [8] (Figure 5A, 5B and 5C). After the inoculation with *B. cinerea*, the control lines dried and died, while most of the *AaERF1*-overexpression lines were growing well (Figure 6). The results showed that overexpression of *AaERF1* could increase the resistance to *B. cinerea* in Arabidopsis.

Six days after inoculated with *B. cinerea*, nearly all the *AaERF1*i transgenic *A. annua* showed symptoms of infection, while the control plant were growing well (Figure 7B). Yu *et al.* showed that AaERF1 could directly bind to the CBF2 and RAA motifs present in both *ADS* and *CYP71AV1* promoters [17]. In the *AaERF1*i transgenic lines, as a result of reduced *ADS* and *CYP71AV1* gene expression, the contents of artemisinin and artemisinic acid were decreased to 76–58% and 55–30% of the wild-type level, respectively [17]. For large amounts of specialized metabolites are considered briefly and related to demonstrated or presumed roles in plant defense [38], [39], the reduction of artemisinin and artemisinic acid may result in reduction of the resistance to *B. cinerea* in *A. annua*. From the above results, we conclude that *AaERF1* is a positive regulator of the resistance to *B. cinerea* in *A. annua*.

In conclusion, the promoter of *AaERF1* was cloned by genomic walking and the GUS staining results of *AaERF1* promoter-GUS transgenic *A. annua* showed that *AaERF1* is ubiquitously expressed in *A. annua*. The expression of *AaERF1* can be induced vigorously by MeJA, ethephon and wound treatments, implying that *AaERF1* may activate some of the defense genes via the JA and ET signaling pathways of *A. annua*. Electrophoretic mobility shift assay (EMSA) and yeast one-hybrid results showed that AaERF1 was able to bind to the GCC box *cis*-acting element *in vitro* and in yeast. The overexpression of *AaERF1* could enhance the expression levels of *Chi-B* and *PDF1.2* and increase the resistance to *B. cinerea* in the 35S::*AaERF1* transgenic Arabidopsis. The down-regulated expression level of *AaERF1* evidently reduced the resistance to *B. cinerea* in *A. annua*. These data suggested that *AaERF1* could not only regulate the artemisinin biosynthetic pathway, but also play important roles as a positive regulator of the resistance to *B. cinerea* in *A. annua*.

## Materials and Methods

### Plant Materials

The seeds of *A. annua* were obtained from the School of Life Sciences, Southwest University in Chongqing, P.R. China. The plants of *A. annua* were grown in a greenhouse. *Arabidopsis thaliana* ecotype Columbia-0 was used in this study and grown under 16 h light (70 mmol m^-2^s^-1^) and 8 h dark cycle at 22°C. Different tissues of *A. annua* and Arabidopsis plants were collected for RNA extraction using plant RNA isolation reagent (Tiangen Biotech, Beijing) following the manufacturer’s instructions. The concentration of the purified RNA was quantified with a nucleic acid analyser (Nanodrop-1000, Nano).

### Isolation and Analysis the *AaERF1* Promoter

The upstream region of *AaERF1* was amplified from the genomic DNA using the Genome Walker Kit (Clontech, Canada). The *AaERF1*-specific primers (AaERF1-sp1, AaERF1-sp2, Adaptor Prime1 and Adaptor Prime2) were used following the manufacturer's recommended procedures. The products amplified from the final reaction products were electrophoresed in 1% agarose gel, and a 1543 bp fragment was eluted from the gel and cloned into the pMD18-T-simple vector. The insert DNA was sequenced by Shenzhen Genomics Institute. The sequence obtained was searched for putative *cis*-acting elements previously characterized using the PlantCare software (http://bioinformatics.psb.ugent.be/webtools/plantcare/html/).

### β-galactosidase (GUS) Expression in Transgenic *A. annua*

To generate the *AaERF1* promoter-GUS construct, the 5′-flanking DNA of the *AaERF1* coding region was amplified with *AaERF1-PF* and *AaERF1-PR*. The 1.5 kb PCR fragment was cloned into the pCAMBIA1391Z vector for sequence confirmation. The construct was transformed into *A. annua* plants as described previously [40]. Histochemical staining for GUS activity in transgenic plants was performed as the protocol described previously [41]. Plants transformed with pCAMBIA1391Z were used as a parallel negative control.

### Hormonal and Stress Treatments

*A. annua* plants grown in MS medium for 2 weeks were treated with solutions of 100 µM MeJA (Sigma Aldrich, USA) and 500 µM Ethephon. Ethephon, an ethylene releaser, was used as ethylene replacement [42]. Since ethephon on hydrolysis releases ethylene and phosphorus, therefore the disodium hydrogen phosphate buffer (Na_2_HPO_3_, 5 mM) was prepared for the decomposition of ethephon. 100 mL ethephon solution was mixed with 100 mL disodium hydrogen phosphate (5 mM). Ten plants were transfered to new petri dishes and pooled for each treatment. For wound induction, the same mixed plants of *A. annua* were cut some 2–3 mm nicks and kept at 22°C under humidified conditions. For the hormonal treatments, all the plants were collected before treatment (0 h) and at different time point after treatment (1 h, 3 h, 6 h, 12 h, 24 h) for the gene expression analysis. For the wound treatments, a time point of 0.5 h was added.

### Expression Pattern Analysis of *AaERF1* by RT-Q-PCR

Expression patterns of *AaERF1* in response to hormonal and stress treatments of *A. annua* were analyzed using the RT-Q-PCR method. The expression levels of *AaERF1*, *Chi-B* and *PDF1.2* in *AaERF1*-overexpressing Arabidopsis were also analysed by this method. All RNA samples were digested with DNase I (RNase-free) prior to use. Aliquots of 0.4 µg total RNA were employed in the reverse transcriptase reaction using random hexamer primers for the synthesis of first strand cDNA. The amplification reactions of qRT-PCR were performed on an iCycler iQTM Real-Time PCR Machines (Bio-Rad, Watford, UK) with gene-specific primers (Table S1), and the SYBR ExScript RT-PCR kit (Takara, Shiga, Japan) protocol to confirm changes of gene expression. The RT-Q-PCR cycling was performed at 37°C (3 min), 95°C (4 min), 40 cycles at 95°C (15 s), 56°C (40 s), 72°C (40 s). The expression of all genes was normalized against the expression of the endogenous control gene (*Actin*). Values displayed are mean fold change as calculated by the 2^-ΔΔCT^ method. All experiments were repeated three times.

### Comparative and Bioinformatic Analysis

Based on the sequence of *AaERF1* (JN162091), one pair of primers (*AaERF1-*F and *AaERF1-*R) were designed, synthesized and used to amplify the full-length sequence of *AaERF1* from *A. annua*. Comparative and bioinformatic analysis of *AaERF1* were carried out online at the websites, http://www.ncbi.nlm.nih.gov and http://cn.expasy.org. Sequence analysis was performed using DNAMAN software (Lynnon Biosoft, USA) and Vector NTI software (Invitrogen). The phylogenetic analysis of AaERF1 protein and ERF proteins from other species was carried out by alignment with CLUSTAL X (1.81) using default parameters. A phylogenetic tree was constructed by neighbor-joining method using software MEGA version 3.1 [43], [44].

### Electrophoretic Mobility Shift Assay

The *AaERF1* cDNA sequence was cloned into the *Eco*RI and *Pst*I sites of the pMAL-C2 vector to produce a MBP-AaERF1 fusion construct (New England BioLabs). The sequenced pMAL-C2-AaERF1 construct was introduced into *E. coli* BL21 for expression. Fusion proteins were expressed in BL21 cells by adding 0.5 mM IPTG to culture medium for 7 h at 28°C and purified using amylose resin (New England BioLabs). The purified recombinant protein was quantified using the Bradford assay (2-D Quant Kit, Amersham Biosciences Corp., San Francisco, CA, USA). The 3′ end biotin-labelled oligonucleotides for the GCC box and the mutated mGCC box were synthesized (Sangon) and equimolar pairs were annealed using the protocol provided by Sigma (Table 1). The binding reaction, gel preparation and electrophoretic mobility-shift assay (EMSA) were performed following the protocol of Zhang *et al.* [45].

### Yeast one-hybrid

To analyze the GCC-binding activity of AaERF1 protein, the entire encoding regions of *AaERF1* was fused into the *Bam*HI-*Xho*I sites of the activation domain of the pB42AD vector (pAD). Reporter vectors containing the 4 × GCC lacZ was prepared and integrated into the yeast strain EGY48, inserted upstream of the minimal promoter element (MP) and the reporter gene lacZ existed in the vector p178. Vectors were introduced into yeast strain through LiAc mediated transformation method (Clontech, Shanghai, China). The cells were grown on tryptophan- and uracil- deficient SD medium for 2–3 days at 30°C, and then transferred to 5-bromo-4-chloro-3-indolyl-beta-Dgalactopyranoside (X-gal) containing plates for color change observation [46].

### Overexpression in *A. thaliana*

The full-length *AaERF1* coding sequence was amplified with primers AaERF1-F and AaERF1-R by Platinum PrimeSTAR HS DNA polymerase (Takara, Shiga, Japan) and subcloned into pMD18-T simple vector. The pMD18-T-AaERF1 vector was digested by *Sac*I and *Bam*HI. The full-length ORF of *AaERF1* was cloned into the *Bam*HI and *Sac*I sites of the pCAMBIA2300+ vector under the 35S promoter to generate pCAMBIA2300-35S::*AaERF1*::NOS. The construct was transferred into *Agrobacterium tumefaciens* GV3101, and then introduced into *A. thaliana* (ecotype Columbia) plants using the floral dip method [47]. Transgenic plants were selected on MS plates containing 50 µg/mL kanamycin. PCR was performed to verify the transgenic status of the screened plants.

### RNAi in *A. annua*

The 201 bp fragment of *AaERF1*, corresponding to *AaERF1* cDNA from nucleotides 179–378, was cloned from *A. annua* by RT–PCR. In RT–PCR, AaERF1i-F (with *Xba*I and *Xho*I sites) and AaERF1i-R (with *Bam*HI and *Hin*dIII sites) were used as the forward and reverse primers, respectively. The amplified fragment was cloned into pMD18-T simple vector and sequenced. After confirmation by sequencing, the fragments were forwardly and reversely placed on the two end sides of the GUS intron in pBluescript SK+ to construct the hp structure. Then the expression cassette was excised with *Sac*I and *Kpn*I from pBluescript SK+ containing the *AaERF1* hp structure and ligated into the expression vector pCAMBIA2300+ to get the final hp *AaERF1*i-containing vector pCAMBIA2300:: p35S-hairpin *AaERF1*-nos. pCAMBIA2300 vector containing only nptII (neomycin phosphotransferase gene conferring resistance to kanamycin) was used as the control vector in transformation. The pCAMBIA2300:: p35S-hairpin *AaERF1*-nos and pCAMBIA2300+ vectors were then transferred into *A. tumefaciens* strain EHA105 by a conventional freezing and thawing method, and the resulting strains were used in the transformation of *A. annua*. The transformation of *A. annua* was performed following the protocol of Zhang *et al.* [40]. All the primers used in this study are in the Table S1.

### Pathogen Infections

*B. cinerea* was grown on potato dextrose agar plates for about 2 weeks at 26°C. Spores were collected from the cultures and washed twice with sterile water. Washed spores were suspended by 10 mL of sterile water, and the suspension was filtered through miracloth to remove mycelia. Four-week-old plants were spray with spore suspensions (2 × 10^5^ spores mL^-1^) and maintained under high humidity. Disease development was observed over the following 6 d. Inoculated plants were scored based on the presence of any disease symptoms after 4 d inoculation with *B. cinerea*, including chlorosis of the leaves, curling and necrosis of the leaves. For each of the control and transgenic plant lines, three biological replicates were performed in parallel.

## Supporting Information

Figure S1 Gus-staining of transgenic *A. annua* using the pCAMBIA1391Z empty vector plasmid.
(TIF)

Figure S2 **Comparison of AP2/ERF domain sequences and dendrogram of ERF proteins.** A. Amino acid alignment of the AP2/ERF domains between AaERF1 and ERF proteins. Highly conserved residues in all the sequences are indicated in white with black background and only partially conserved residues in ERF proteins are showed in black with grey background. One α-helix and three β-sheets are marked above the corresponding sequences. The YRG and RAYD elements are indicated with solid lines below the consensus sequence. B. A phylogenetic tree of the ERF proteins was constructed. Alignments were made in Clustal X using the default parameters. Accession numbers for the AP2/ERF proteins used are as follows: AtERF1, AF076277; AtERF2, NM124093; AtERF3, XP002894264; AtERF4, NM112384; AtERF5, NM124094; AtERF6, Q8VZ91; AtERF7, NM112922; AtERF8, Q9MAI5; AtERF9, Q9FE67; AtERF10, Q9ZWA2; ERF1, AAD03545; ORA59, NM100497; LeERF1, Q84XB3; TaERF3, EF570122; NtERF1, Q40476;ORCA3, EU072424; GmERF3, EU681278; AaERF1, JN162091).
(TIF)

Figure S3 **Analysis of transgenic** ***A.annua*** **plants by PCR.** A. PCR analysis of 35S forward primer and *AaERF1* reverse primer in *AaERF1*-RNAi transgenic plants. M: DNA size marker DL2000, V: empty-vector transgenic *A. annua*, C: water control, P: positive control. B. PCR analysis of 35S-forward primer and the reserse prmer of kanamycin-resistant gene in *AaERF1*-RNAi transgenic plants.
(TIF)

Table S1 Primers used in this study.
(DOC)
