# Supplementary figures and images for: AaERF1 Positively Regulates the Resistance to Botrytis cinerea in Artemisia annua
Source: PLoS One. 2013 Feb 28;8(2):e57657. doi: 10.1371/journal.pone.0057657 (PMC3585223; doi:10.1371/journal.pone.0057657)

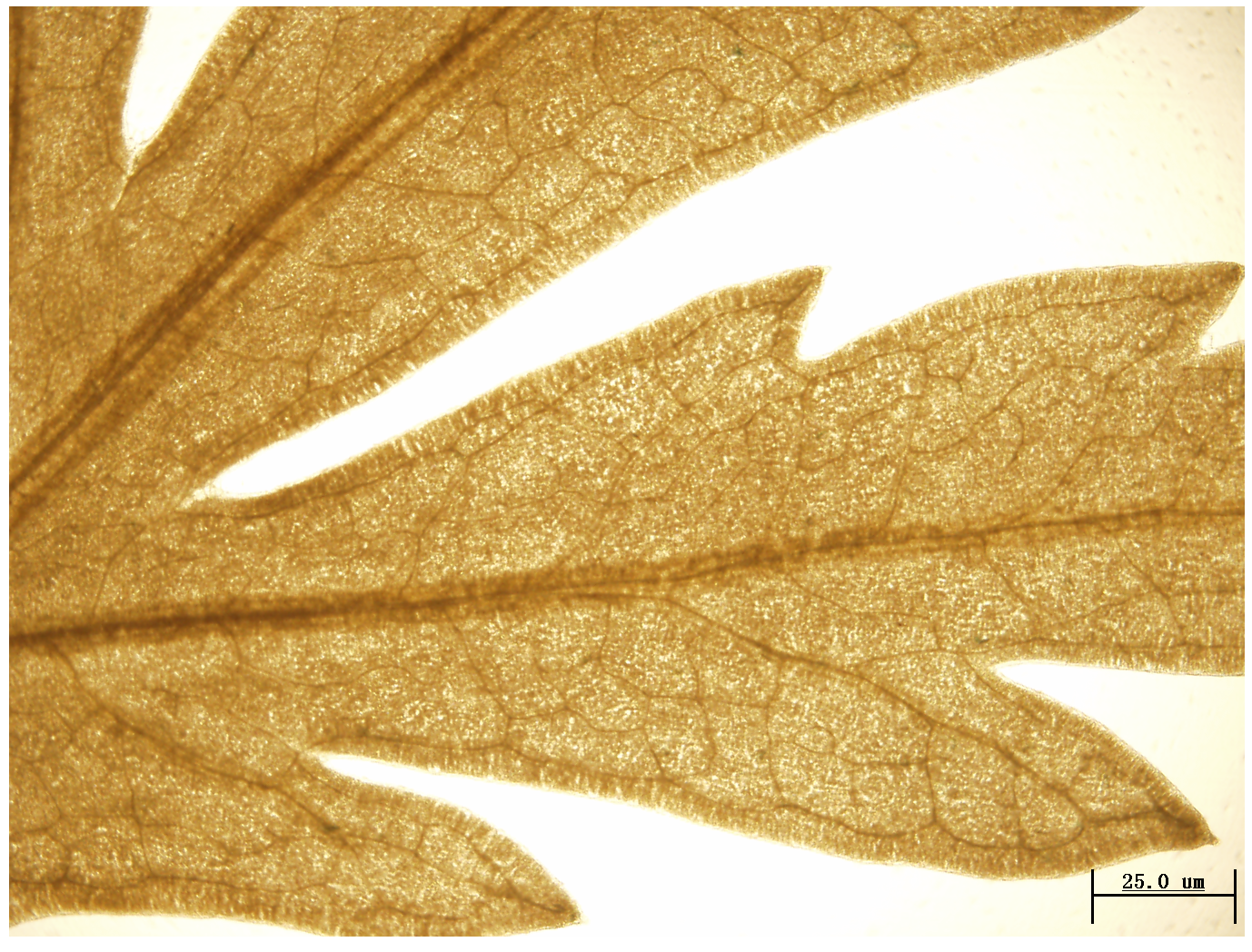

Supplement: Figure S1 — Gus-staining of transgenic A. annua using the pCAMBIA1391Z empty vector plasmid. (TIF) [file pone.0057657.s001.tif]

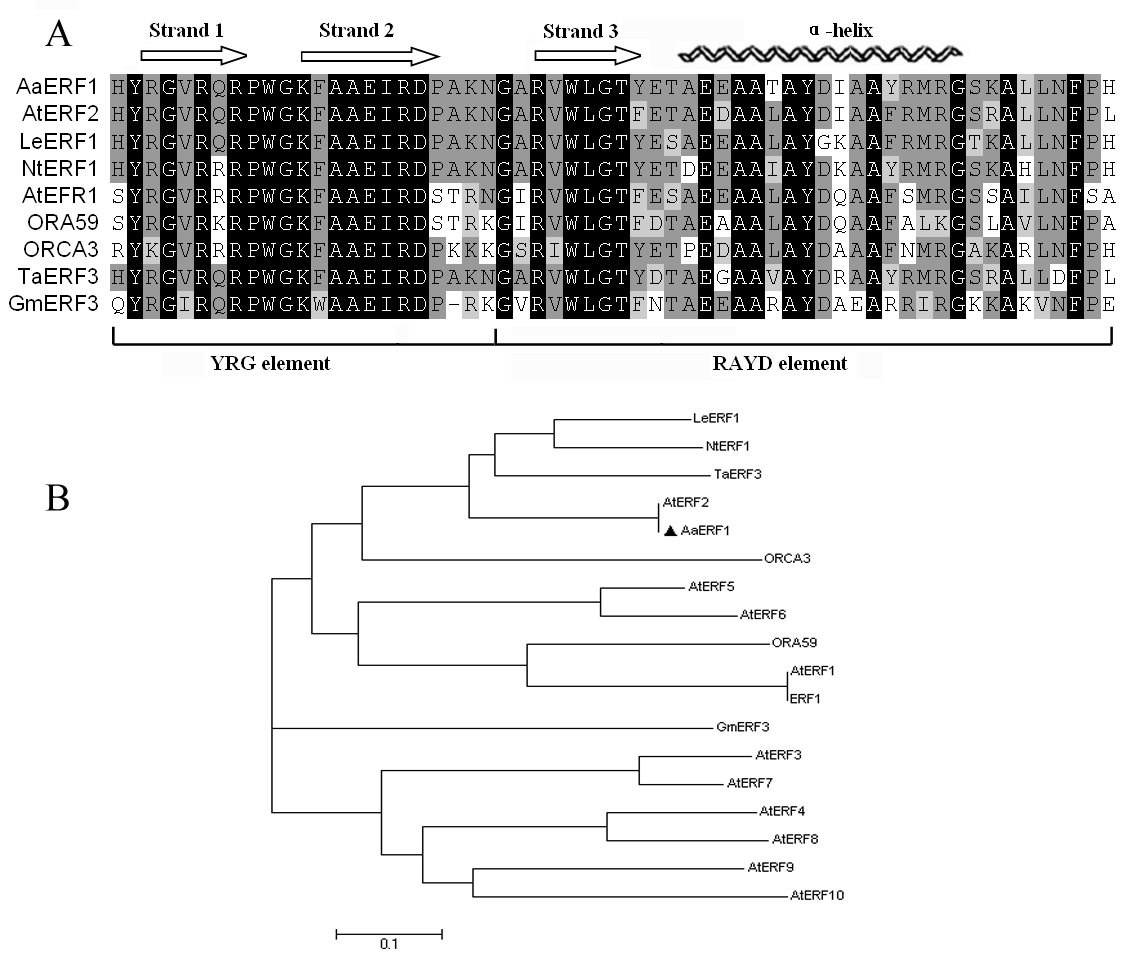

Supplement: Figure S2 — Comparison of AP2/ERF domain sequences and dendrogram of ERF proteins. A. Amino acid alignment of the AP2/ERF domains between AaERF1 and ERF proteins. Highly conserved residues in all the sequences are indicated in white with black background and only partially conserved residues in ERF proteins are showed in black with grey background. One α-helix and three β-sheets are marked above the corresponding sequences. The YRG and RAYD elements are indicated with solid lines below the consensus sequence. B. A phylogenetic tree of the ERF proteins was constructed. Alignments were made in Clustal X using the default parameters. Accession numbers for the AP2/ERF proteins used are as follows: AtERF1, AF076277; AtERF2, NM124093; AtERF3, XP002894264; AtERF4, NM112384; AtERF5, NM124094; AtERF6, Q8VZ91; AtERF7, NM112922; AtERF8, Q9MAI5; AtERF9, Q9FE67; AtERF10, Q9ZWA2; ERF1, AAD03545; ORA59, NM100497; LeERF1, Q84XB3; TaERF3, EF570122; NtERF1, Q40476;ORCA3, EU072424; GmERF3, EU681278; AaERF1, JN162091). (TIF) [file pone.0057657.s002.tif]

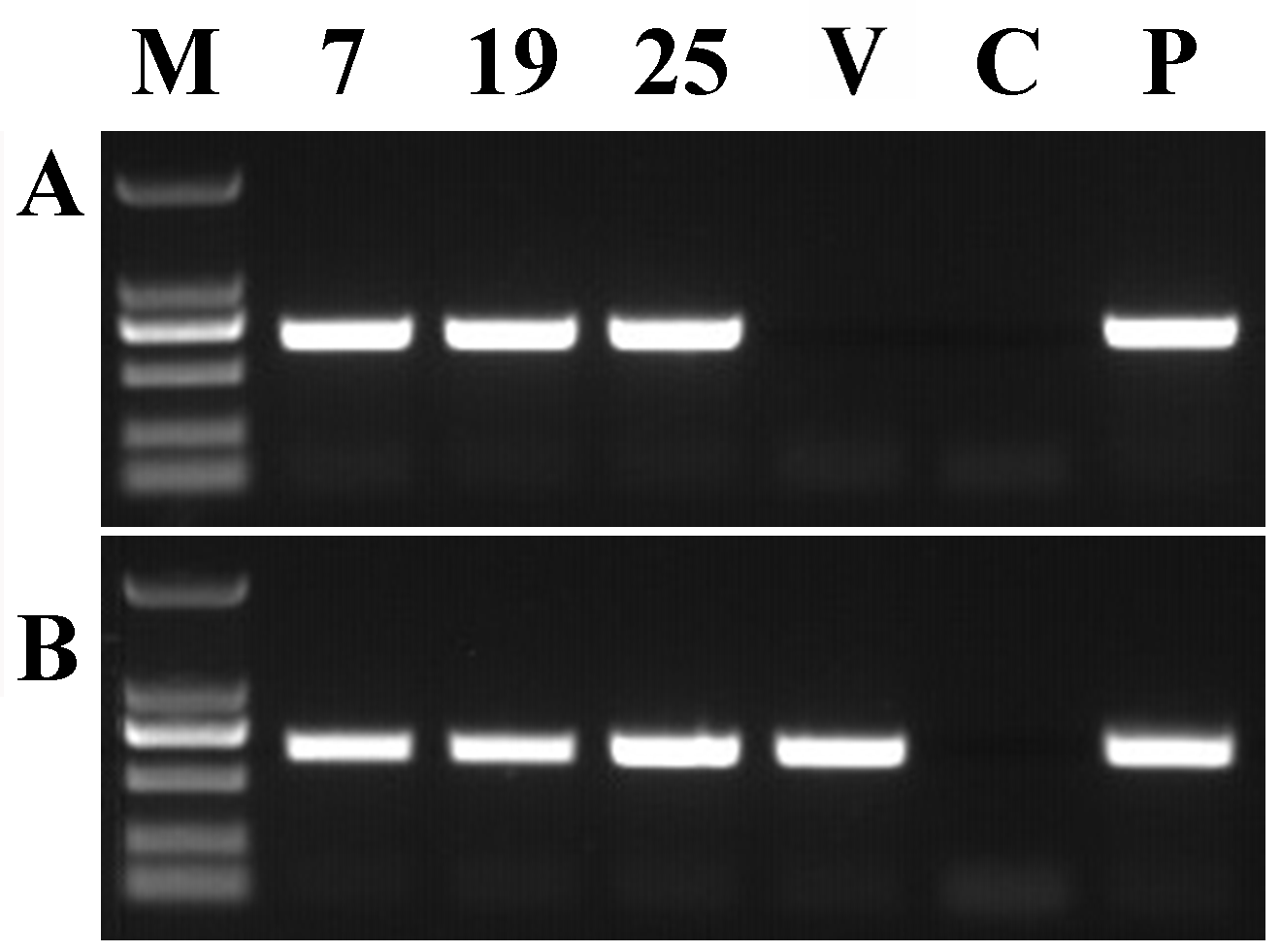

Supplement: Figure S3 — Analysis of transgenic A.annua plants by PCR. A. PCR analysis of 35S forward primer and AaERF1 reverse primer in AaERF1-RNAi transgenic plants. M: DNA size marker DL2000, V: empty-vector transgenic A. annua, C: water control, P: positive control. B. PCR analysis of 35S-forward primer and the reserse prmer of kanamycin-resistant gene in AaERF1-RNAi transgenic plants. (TIF) [file pone.0057657.s003.tif]
