# Supplementary material for: AaERF1 Positively Regulates the Resistance to Botrytis cinerea in Artemisia annua
Source: PLoS One. 2013 Feb 28;8(2):e57657. doi: 10.1371/journal.pone.0057657 (PMC3585223; doi:10.1371/journal.pone.0057657)
Supplement: Table S1 — Primers used in this study. (DOC) [file pone.0057657.s004.doc]

**Supplementary Table**

**Supplement table S1** Primers used in this study

| *Primers Purpose Primer Sequence(5’-3’)* |
| --- |
| AaERF1-F Clone CCGGATCCATGATGCAAATGCCTTCGTT  AaERF1-R Clone CCGAGCTCTTAACCACTAACGGCTTCAC  Adaptor Prime1 Promoter clone GTAATACGACTCACTATAGGGC  Adaptor Prime2 Promoter clone ACTATAGGGCACGCGTGGT  AaERF1-sp1 Promoter clone TAGGATGCGTGGCTCAAAGGCT  AaERF1-sp2 Promoter clone CGCCAAAAGGGAAGCATTATCG  AaERF1-PF Vector contruction CCCTGCAGGAGTTGGAATCGTGTTA  AaERF1-PR Vector contruction CCGAATTCTTTTAAAATATGGGTTTT  AaERF1-pMAL-F Clone CCGAATTCATGATGCAAATGCCTTCGTT  AaERF1-pMAL-R Clone CCCTGCAGTTAACCACTAACGGCTTCAC  AaERF1-RT-F Q-PCR GTGATGCCGTTAGTGTTGGATGGACT  AaERF1-RT-R Q-PCR CGGCGCTGCCACTGATGT  Actin-F Q-PCR CCAGGCTGTTCAGTCTCTGTAT  Actin-R Q-PCR CGCTCGGTAAGGATCTTCATCA  PDF1.2-F Q-PCR TTGCTGCTTTCGACGCA  PDF1.2-R Q-PCR TGTCCCACTTGGCTTCTCG  CHI-B-F Q-PCR ATCAGCGCTGCAAAGTCCTTC  CHI-B-R  Q-PCR GTGCTGTAGCCCATCCACCTG  At-ACTIN-F Q-PCR AGTGGTCGTACAACCGGTATTGT  At-ACTIN-R Q-PCR GAGGAAGAGCATTCCCCTCGTA  GCC box EMSA CATAAGAGCCGCCACT  mGCC box EMSA CATAAGATCCTCCACT  P178-F4*GCC Vector contruction TCGAGAAGCTTGATCAGCCGCCGGATC  GATCAGCCGCCGGATCGATCAGCCGCC  GGATCGATCAGCCGCCGGATCC  P178-R4*GCC Vector contruction TCGAGGATCCGGCGGCTGATCGATCCG  GCGGCTGATCGATCCGGCGGCTGATCG  ATCCGGCGGCTGATCAAGCTTC  AaERF1- pB42F Vector contruction CCGAATTCATGATGCAAATGCCTTCGTT  AaERF1- pB42R Vector contruction CCCTCGAGTTAACCACTAACGGCTTCAC |
